# Carrot-Induced Systemic Reaction: A Unique Presentation of Pollen-Food Allergy Syndrome in a Young Boy

**DOI:** 10.3390/children10111817

**Published:** 2023-11-15

**Authors:** Weronika M. Balas, Joanna Strzelecka, Aleksandra Godyńska, Adam J. Sybilski

**Affiliations:** 1 Department of Paediatrics and Allergology, The National Institute of Medicine of the Ministry of the Interior and Administration, 02-507 Warsaw, Polandaleksandra.godynska@cskmswia.gov.pl (A.G.); adam.sybilski@cskmswia.gov.pl (A.J.S.); 2 2nd Department of Paediatrics, Centre of Postgraduate Medical Education, 01-813 Warsaw, Poland

**Keywords:** pollen-food allergy syndrome, food allergy (hypersensitivity), anaphylaxis

## Abstract

Pollen-food allergy syndrome (PFAS) is a common IgE-mediated allergic condition resulting from cross-reactions between pollen and plant food allergens, primarily those in the PR-10 subfamily. Mostly symptoms are limited to the mouth and throat causing oral allergy syndrome (OAS). Systemic reactions are extremely rare. We report an 11-year-old boy who experienced a unique anaphylactic reaction after consuming raw carrot juice. The patient exhibited symptoms within one minute, including abdominal pain, facial and eyelid swelling, dyspnea, a macular rash, choking sensation and drowsiness. Desloratadine alleviated these symptoms, and as his overall condition improved rapidly, there was no need for adrenaline administration. Carrot-specific IgE levels in the patient’s serum were as follows: Dau c: 40.63 kUA/L and Dau c1: 31.5 kUA/L. He had previously been diagnosed with seasonal allergic rhinoconjunctivitis. The high degree of similarity among allergen components within the PR-10 subfamily contributed to cross-reactivity between birch pollen and carrots. It is important to remember that PFAS can manifest systemically, with symptoms ranging from mild skin itching to potentially fatal consequences. This highlights the need for healthcare professionals to be extra cautious and aware of this possibility, especially since carrots are commonly found in a wide range of dishes and snacks.

## 1. Introduction

Pollen-food allergy syndrome (PFAS) is a common IgE-mediated allergic disease caused by a cross-reaction between pollen and plant food allergens [1]. The pathogenesis of PFAS is related to a respiratory allergy to plant pollen and subsequent cross-reaction between pollen and homologous epitopes of proteins contained in foods of plant origin [2,3].

The characteristic clinical picture of PFAS is most often isolated symptoms in the mouth and throat that appear immediately after ingestion of food, which is called oral allergy syndrome (OAS) [4]. PFAS symptoms are generally limited to the mucous membranes of the mouth and throat and appear immediately or within 5 to 10 min after eating fresh fruits, vegetables, nuts, legumes and seeds. These usually include itching of the lips, mouth and throat, paraesthesia, angioedema of the oral mucosa, lips, tongue, palate and throat, itching of the ears, hives and a feeling of tightness in the larynx which may cause hoarseness. They generally last from a few minutes to half an hour. Systemic reactions such as nausea, abdominal pain, diarrhoea, rhinitis, shortness of breath, skin rash, urticaria, angioedema or hypotension and anaphylaxis are rare to extremely rare (1–2% for anaphylaxis) [5,6].

PR-10 proteins are the main cause of PFAS occurrence with oral symptoms in northern and central Europe, where an inhalant allergy to birch (Bet v 1) and alder pollen predominates, and symptoms are most often caused by fruits from the Rosaceae family containing PR-10 (e.g., apple [Mal d 1], cherry [Pru av 1], apricot [Pru ar 1], pear [Pyr c 1]), or vegetables from the Apiaceae family (e.g., carrot [Dau c 1], celery [Api g 1]) and hazelnut (Cor a 1) [2,7].

PR-10 proteins are labile and sensitive to heat and digestion; therefore, patients often tolerate cooked fruits or vegetables, which in raw form cause OAS [8,9].

In this case report, we want to present an anaphylactic reaction as a manifestation of PFAS syndrome.

## 2. Detailed Case Description

**Figure 1:**
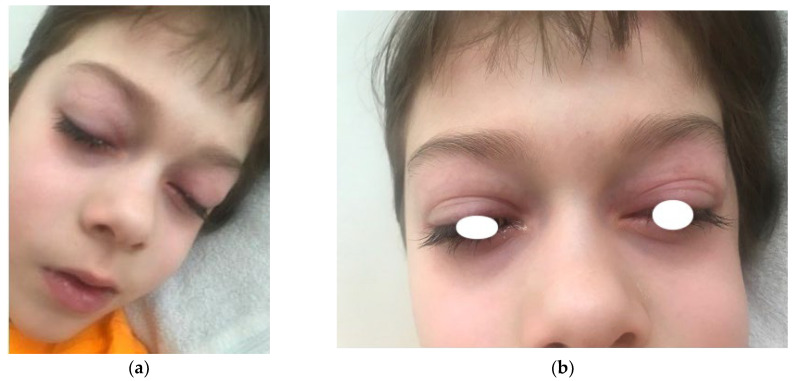
Images of the face of the present patient after 4 doses of desloratadine (**a**,**b**).

An 11-year-old boy had experienced an anaphylactic reaction after consuming approximately 200 mL of store-bought fresh juice containing 100% raw carrots. The patient exhibited symptoms within one minute, including abdominal pain, facial and eyelid swelling, dyspnea, a macular rash, choking sensation and drowsiness (Figure 1). Four doses of desloratadine (10 mg) alleviated these symptoms, and as his overall condition improved within minutes, there was no need for adrenaline administration. In cases of anaphylaxis, healthcare professionals typically administer epinephrine as the initial treatment, but in this boy’s situation, the parents administered antihistamines at home. After antihistamines, the boy’s symptoms alleviated, causing his parents to believe that there was no need for immediate medical care. This episode marked his second anaphylactic reaction. In preschool age, he experienced a similar reaction after consuming a cookie containing chocolate, hazelnuts and almonds. The earliest symptoms were shortness of breath and abdominal pain, followed by urticarial blisters on the face and mouth along with swelling of the face and eyelids. At that time, his symptoms were also relieved with four doses of desloratadine. Both reactions occurred during the birch pollen season (I—Spring, 5 years; II—April, 11 years).

He was born from a second pregnancy, delivered naturally at 40 weeks of gestation. At the age of six, the patient experienced his first allergic symptoms, including eyelid swelling and itching, which were relieved with desloratadine. These allergic symptoms appeared after consuming paprikash (ground fish meat with rice, onions, tomato paste, in vegetable oil, with spices and salt) or pumpkin seeds. Furthermore, he experienced sneezing after consuming walnuts, peanuts and peanut butter, as well as a macular facial rash after consuming peaches and plums during the summer. Importantly, there is no history of asthma or allergies related to animals or dust. He was diagnosed with vasomotor rhinitis and allergic rhinoconjunctivitis, with symptoms worsening from February to June. His family history was positive for allergies: the patient’s grandmother has a history of food allergies, and the patient’s uncle (mother’s brother) has a history of bronchial asthma. To manage his symptoms, the patient currently takes cetirizine dihydrochloride, uses eye drops containing olopatadine or ketotifen, and uses a nasal spray with mometasone furoate during symptomatic periods. The boy has adrenaline at home for use in case of an emergency. The patient was instructed to maintain a diet with elimination of products that cause allergic symptoms, including carrots and nuts. In addition, prevention of a dust mite allergy was recommended, i.e., frequent vacuuming and airing of rooms, removal of curtains, carpets and rugs.

Physical examination revealed no apparent abnormalities. The serum carrot-specific immunoglobulin E levels were as follows: Dau c: 40.63 kUA/L [reference range: 0.00–0.35 kUA/L] and Dau c 1: 31.5 kUA/L [reference range: 0.00–0.35 kUA/L]. Additionally, he tested positive for grass, tree and weed pollens, mites, microorganism-based allergens, plant-based food allergens, and animal-based allergens [Table 1]. Total IgE was 3518 kU/L. Levels of IgE specific for 295 allergens were determined using Allergy Xplorer ALEX2 (Macro Array Diagnostics-MADX, Vienna, Austria).

## 3. Discussion

This case represents an extremely rare occurrence of an IgE-mediated anaphylactic reaction, which manifested as a part of pollen-food allergy syndrome (PFAS). It occurred after the consumption of allergen components from the PR-10 subfamily. The main allergen of this subfamily is Bet v 1, which is found in birch pollen. Allergic reactions to Bet v 1 typically manifest as rhinoconjunctivitis, as previously diagnosed in this patient. The combination of the allergenic component of birch pollen with IgE antibodies present on the surface of mast cells in the nasal mucosa or basophils in the peripheral blood causes the release of histamine and other inflammatory mediators. This contributes to symptoms like sneezing, itching, leakage of watery nasal discharge and later nasal congestion.

The high degree of similarity among allergen components within the PR-10 subfamily contributes to birch pollen cross-reactivity with various food allergens. Common examples of these cross-reactions include birch–apple, birch–celery, as well as birch–carrot [10]. It is estimated that up to 60% of children and adolescents with food allergies also have co-existing inhalant allergies [11]. However, they very rarely contribute to anaphylactic reactions. Soy products (Gly m 4), hazelnuts (Cor a 1.04) and celery (Api g1) are the most common sources of allergen components responsible for these reactions [10]. Anaphylactic reactions are potentially fatal allergic reactions involving multiple organs, triggered by the release of chemical mediators from mast cells and basophils. It typically causes more than one of the following: an itchy rash, throat closing due to swelling that can obstruct or stop breathing; severe tongue swelling that can also interfere with or stop breathing; and shortness of breath, vomiting, lightheadedness, loss of consciousness, and low blood pressure [11]. These listed symptoms result from distinct pathophysiological mechanisms: smooth muscle contraction, contributing to abdominal pain and bronchospasm; increased vascular permeability, leading to angioedema, hypotension, and urticaria; and mucous membrane swelling, which can manifest as nasal congestion or laryngeal swelling. A systematic review of studies conducted in Europe found that the lifetime incidence rate of anaphylaxis ranged from 1.5 to 7.9 per 100,000 person years [11]. It is most frequently triggered by food, drugs, stinging insects and latex. The percentage of anaphylaxis cases attributed to food allergens varies between 0.4% and 39.9%, with eggs, milk, nuts, fish, fruit and sesame being the most common culprits [11]. The primary treatment of anaphylaxis is epinephrine injection into a muscle then placing the person “in a reclining position with feet elevated to help restore normal blood flow”. Additional doses of epinephrine may be required. Other measures, such as antihistamines and steroids, are complementary [11].

Reported cases of a severe allergic reaction to carrot are relatively rare. In 1941, Vickers reported seven cases of dermatitis of the hands in kitchen workers attributed to contact with carrots [12]. In 1996, Gómez et al. presented the case of a female cook who experienced allergic rhinoconjunctivitis and contact urticaria in her hands, accompanied by severe itching when handling raw carrots [13]. However, after heat treatment, carrots no longer induced these symptoms. In 2022, Hirai et al. showed an interesting patient who experienced an anaphylactic reaction after consuming cooked carrots immediately after playing—physical activity [14]. Although the patient consumed the cooked allergen, it caused an intensified systemic reaction. Physical activity was a contributing factor that led to anaphylaxis due to increased allergen bioavailability. The term that describes this phenomenon is food-dependent exercise-induced anaphylaxis (FDEIA). Cofactors, which are factors that co-occur and collaboratively contribute to the reaction, play a significant role, particularly in adolescents, in the development of anaphylaxis.

In 2023, Schiappoli et al. described the case of a man who twice experienced anaphylactic shock, both times due to PR-10 proteins. The first occurrence was after consuming a carrot and celery appetizer, and the second incident happened after consuming chocolate ice cream containing a hidden carrot allergen [15]. The researchers highlighted the information that the patient had been suffering from seasonal allergic rhinitis for over 10 years. It is worth noting that reactions in our patient, both to carrots and to cookies containing nuts, occurred during the birch pollen season. It is commonly observed that symptoms intensify during the birch pollen season or shortly after its conclusion. In some cases, as seen in this patient, it may even lead to an anaphylactic reaction.

Another potential adverse reaction associated with the consumption of raw carrot juice is methemoglobinemia, a condition that can also lead to shortness of breath. Well-fertilized root vegetables like carrots can accumulate nitrates, particularly in low-processed or improperly stored products. Spoiled raw carrot juice, stored without refrigeration, can promote the conversion of nitrate to nitrite, potentially leading to severe nitrite poisoning and subsequent methemoglobinemia. Signs and symptoms of methemoglobinemia typically include cyanosis, shortness of breath, fatigue, headache, mental confusion and a rapid heart rate. These clinical manifestations may vary in severity depending on the degree of methemoglobinemia. For the management of methemoglobinemia, methylene blue is the primary treatment option, with partial exchange transfusion considered in severe cases [16]. In the case presented, it is crucial to emphasize that the juice consumed was fresh. Furthermore, a meticulous examination of the patient’s symptoms and specific IgE levels unquestionably align with a diagnosis of anaphylactic shock, definitively excluding the possibility of methemoglobinemia.

The patient described in this paper had earlier eaten both cooked and raw carrots without symptoms. We hypothesize that the severity of PFAS symptoms correlates with the concentration of birch pollen in the air, with these symptoms aggravating during the pollen season and shortly afterward [10]. In an article by Yasudo et al., it is proposed that PFAS could potentially be a part of the allergic march [17]. The study found that at the age of five, IgE sensitization to Bet v1 was observed in 2.2% of participants, and this percentage increased to 13.9% by the age of nine. This increase in IgE level was identified as a risk factor for PFAS in adolescence. It is plausible that the boy is experiencing this phenomenon, progressing from pollinosis to a unique systemic reaction, as a manifestation of PFAS [17]. The patient reports allergy symptoms to peaches and plums, for which he has no sIgE detected. In the case of an allergy without specific IgE antibodies, it is possible that the allergy is mediated by a different immune mechanism [11].

In the case of the patient described here, in vitro diagnostic procedures revealed elevated levels of IgE specific to various food and inhalatory allergens. The patient’s serum contained specific IgE (sIgE) that showed very strong cross-reactivity with PR-10 allergens and secretory immunoglobulins and weak cross-reactivity with storage proteins, lipocalins and NPC 2. Through in vitro diagnostic tests, we detected elevated levels of IgE specific to various food allergens, including celery, soy, carrots, strawberries, chickpea, peach, walnut, hazelnut, pecan nut, millet, paprika and apples. Furthermore, based solely on these results, it is not possible to differentiate between a clinically significant allergy and asymptomatic sensitization. While the sIgE levels are elevated for several allergens, the reaction to them remains uncertain. This uncertainty arises from the fact that the patient has no exposure to all of them in the home environment.

## 4. Conclusions

An anaphylactic reaction to consuming carrot juice, resulting from cross-allergy with birch pollen Bet v 1, was diagnosed. Individuals with inhalation allergies frequently exhibit typical symptoms of PFAS. Within this group, some patients may also experience systemic symptoms. In any such case, immediate administration of epinephrine is crucial. This paper highlights the need for healthcare professionals to be extra cautious and aware of the possibility of anaphylaxis triggered by carrots, PR-10 proteins, especially since carrots, a versatile ingredient, are commonly found in a wide range of dishes and snacks.

## Figures and Tables

**Table 1:** Specific IgE levels, which were detected in the serum using ALEX2. The PR-10 subfamily is bolded.

		Allergen	sIgE [kUA/L]
**Grass pollen**	Timothy Meadow	Phl p 5.0101	30.25
		Phl p 6	2.68
	Rye pollen	Sec c_pollen	1.9
**Tree pollen**	Acacia	Aca m	1.54
	Alder	Aln g 1	42.39
		Aln g 4	0.19
	Birch	Bet v 1	45.24
	Hazel	Cor a_pollen	17.36
		Cor a 1.0103	33.95
	Beech	Fag s 1	36.62
	Walnut	Jug r_pollen	22.1
	Poplar	Pop n	1.17
	Cypress	Cup s	0.16
	Ash	Fra e	0.23
**Weed pollen**	Amarant	Ama r	1.19
	Russian thistle	Sal k	0.34
**Mites**	Dermatophagoides farinae	Def f 2	9.28
	Dermatophagoides pteronyssinus	Der p 2	10.41
	Blomia tropicalis	Blo t 10	0.29
**Microorganism**	Malassezia sympodialis	Mala s 6	0.57
	Alternaria alternata	Alt a 1	0.17
**Plant-Based Food**	Peanut	Ara h 8	20.45
	Soybean	Gly m 4	10.64
	Pea	Pis s	0.11
	Paprika	Cap a	0.19
	Rice	Ory s	0.15
	Millet	Pan m	0.55
	Strawberry	Fra a 1 + 3	35.08
	Apple	Mal d 1	22.17
	Pear	Pyr c	2.81
	Celery	Api g 1	36.34
	Carrot	Dau c	40.63
		Dau c 1	31.5
	Pecan	Car i	0.83
	Hazelnut	Cor a 1.0401	36.72
	Walnut	Jug r 1	2.35
		Jug r 2	0.28
**Animals**	Dog	Can f_Fd1	5.96
		Can f 1	12.95
	Cat	Fel d 1	36.8
		Fel d 7	0.16

## Data Availability

No new data were created or analyzed in this study. Data sharing is not applicable to this article.
